# A Half-Century Analysis of Firearm-Related Mortality Trends in the United States: A Retrospective Study Utilizing National Data (1968–2022)

**DOI:** 10.7759/cureus.74228

**Published:** 2024-11-22

**Authors:** Oluwasegun A Akinyemi, Temitope Ogundare, Terhas Wedeslase, Brandon Hartmann, Eunice Odusanya, Mallory Williams, Kakra Hughes, Edward Cornwell III

**Affiliations:** 1 Health Policy and Management, School of Public Health, University of Maryland, College Park, USA; 2 Surgery, College of Medicine, Howard University, Washington, DC, USA; 3 Psychiatry and Behavioral Sciences, School of Medicine, Boston University, Boston, USA

**Keywords:** firearm-related death, injury prevention and control, suicide, suicide prevention, united states, violence, violence prevention

## Abstract

Background*:* Firearm-related deaths are a substantial public health crisis in America, with studies reporting an increasing rate in the past decade. Effective public health interventions rely on comprehensive information about risk and protective factors.

Aim: This study aims to provide a comprehensive examination of trends in firearm-related deaths over the past 55 years, shedding light on the changing landscape and identifying key risk and protective factors associated with firearm-related deaths in the United States.

Methods: This retrospective study utilizes data from the Centers for Disease Control and Prevention's Web-based Injury Statistics Query and Reporting System (WISQARS) for 1968-2022 to determine trends in firearm-related deaths. A multivariate logistic regression model was employed to identify independent predictors of firearm-related suicides, homicides, and unintentional deaths, exploring intersectionality by introducing interaction terms between race/ethnicity and level of education.

Results: Firearm-related deaths showed a fluctuating but upward trend from 12.0/100,000 persons in 1968 to 14.5/100,000 in 2022, with firearm-related suicides consistently accounting for a significant proportion of firearm-related deaths, from 45.7% in 1968 to 56.1% in 2022, with a peak of 63% in 2013. From the multivariate regression analysis, individuals aged 10-19 years had the highest risk of firearm-related suicides (OR = 3.04, 95% CI = 2.92-3.16) and homicides (OR = 2.87, 95% CI = 2.77-2.97). In addition, White people with higher education (OR = 1.42, 95% CI = 1.40-1.45) had the highest risk of firearm-related suicides, while Black people with lower educational attainment (OR = 6.68, 95% CI = 6.50-6.87) had the highest risk of firearm-related homicides.

Conclusion: Our findings underscore the urgent need for targeted, evidence-driven public health interventions and policies. Primary suicide prevention strategies focusing on means restriction and reshaping perceptions around firearm ownership emerge as critical components. Comprehensive, multidimensional approaches that engage firearm owners and communities and address structural factors are imperative to curbing the multifaceted challenges associated with firearm-related injuries and deaths. Targeted interventions must include individuals aged 10-19 and specifically focus on suicides and homicides in the most relevant demographic segments of the population.

## Introduction

Firearm injuries and deaths are a significant public health problem in the United States, accounting for about 48,830 deaths in 2021 [1]. Compared to other high-income countries, the United States has higher rates of firearm-related deaths, estimated to be about 25 times higher [2]. Globally, in 2016, the United States accounted for 91.7% of women and 98.1% of children killed by firearms [2]. Black and other minority groups are disproportionately affected by firearm-related deaths; Black men are 10-20 times more likely to be killed by firearms, and American Indian and Alaskan Native men are about 3-4 times more likely to die from firearm-related suicides [3]. Over the past decade, there has been a steady but fluctuating increase in firearm-related deaths in the United States, with firearm-related homicides and suicides contributing to the total number. In 2021, firearm-related suicides accounted for 54% of all firearm-related deaths in the United States, while 43% were firearm-related homicides [1]. Firearm-related deaths have an impact on individuals, families, and communities, with an estimated economic burden of $280 billion annually [4]. Firearm-related deaths are also associated with anxiety disorders, substance use disorders, and increased firearm purchasing [3].

Effective prevention strategies depend on the identification of risk factors. The Centers for Disease Control and Prevention proposed a socioecological model as a framework for determining the various levels of risk factors associated with firearm-related violence [4]. Individual-level risk factors related to firearm-related deaths include low socioeconomic status, mental health disorders, substance use disorders, adverse childhood events, and access to firearms [5]. Risk factors at the interpersonal and community levels include the absence of social support, family mental illness, substance use disorder, community-level poverty/unemployment, aspects of the built environment (such as green spaces, concentration of liquor stores, and vacant lots), social factors (such as access to healthcare, including mental health and substance use treatments), and crime levels [5,6]. At the structural/policy levels, structural racism, funding for firearm prevention research, firearm policies, and policies related to health, economics, and education all serve as risk factors for firearm-related deaths [5,6]. These risk factors interact with each other and are dynamic [5].

The passage of the Omnibus Act in 1996 stalled research into firearms and firearm death prevention for more than two decades by significantly limiting funding, leading to a decline in publications related to firearm injuries and deaths [7,8]. In 2013, the National Academy of Medicine and National Research Council outlined five key areas in developing research in firearm-related injuries and deaths, including characteristics of firearm violence, risk and protective factors, intervention and strategies, firearm safety technology, and influence of video games and other media [7]. There is a need for a comprehensive body of evidence to inform policies and design public health interventions to reduce harm from firearms and improve community safety and health [7].

In this study, we provide comprehensive data on trends in firearm-related deaths in the United States over the past 55 years to provide valuable insights into the changing landscape of firearm mortalities. In addition, we aim to determine the risk and protective factors associated with firearm-related suicides and homicides to provide evidence for the design and implementation of public health interventions. We also aim to provide data that can inform effective policies. Most often, firearm research and violence prevention have been highly politicized. However, it is important that, given the significant burden of firearm-related injuries and deaths, we have data to support arguments for proposed interventions. Data-driven policies and interventions are the most effective and cost-effective [9,10].

## Materials and methods

This study utilized data from the Centers for Disease Control and Prevention’s Web-based Injury Statistics Query and Reporting System (WISQARS). It is a national database containing detailed information on fatal injuries. It covers homicides, suicides, deaths of undetermined intent possibly related to violence, law enforcement fatalities (excluding executions), and unintentional firearm-related deaths. This database offers more comprehensive insights compared to other violent death databases. Violent death is defined as those involving intentional physical force against oneself, another person, or a group/community. The database provides extensive data on circumstances leading to deaths, including events preceding the incident and toxicology details, as well as specifics on weapons, injuries, and other incident characteristics. Circumstances reported vary by manner of death, with suicides and undetermined deaths related to mental health history, disclosure of suicidal intent, and precipitating factors like crises or financial issues. Homicide circumstances focus on criminal activity and interpersonal conflicts, while unintentional firearm-related death circumstances relate to the incident context and firearm usage. Data for WISQARS is sourced from law enforcement reports, medical examiner/coroner reports, and death certificates. The database was launched in 2003 with six states but now includes data from all 50 states, the District of Columbia, and Puerto Rico and is updated annually. Population estimates used in WISQARS are generated by the US Census Bureau in collaboration with the CDC’s National Center for Health Statistics (NCHS) [11]. Violent death statistics in the database rely on International Classification of Disease-10th Revision (ICD-10) codes and manner of death information from source documents. Each record includes data on victims and alleged perpetrators associated with the incident, defined as related fatal injuries occurring within a 24-hour period. WISQARS is a comprehensive, publicly accessible database that provides information on injury-related morbidities and mortalities in the United States. The WISQARS violent module was used in the study. It is a restricted-access database that is available upon request. The data extracted for this study were from 1968 to 2022. The study population comprised all recorded cases in the WISQARS database within the specified timeframe.

Outcome variables

The primary outcome variables were categorized into four distinct types of firearm-related deaths: suicides, homicides, unintentional, and undetermined. These categories were based on the manner of death recorded in the database.

Covariates

Covariates included demographic data, such as age (categorized into different groups), gender, race/ethnicity, and education level; mental health disorders, such as depression, dementia, bipolar disorder, and anxiety; and physical health conditions, such as hypertension and obesity. Temporal factors such as the season of the death occurrence were also included in our analysis.

Statistical analysis

The statistical analysis was conducted using STATA 16 (StataCorp LLC, College Station, TX, United States) statistical software. Descriptive statistics are presented using tables and figures. Age was categorized into four groups: 10-19 years, 20-44 years, 45-64 years, and 65 years and older. Education level was categorized into high school education, tertiary (represents those who have a college education), and advanced (represents those with advanced degrees).

A multivariate logistic regression model was employed to identify independent predictors of firearm-related suicides, homicides, and unintentional deaths. Covariates included in the regression model were age, gender, race/ethnicity, education level, mental health disorders, and seasonal factors, based on their known associations with firearm-related mortalities, as indicated by previous research and theoretical frameworks. The model also included interaction terms between race/ethnicity and education level to explore the intersectionality of these factors. A simultaneous entry method was used to fit the variables in the regression model. This method was selected because it allows for the assessment of the effect of each variable while controlling for the other variables and assessing the overall explanatory power of a set of variables without prioritizing any particular one. The analysis excluded missing data, ensuring that only complete cases were used in the final models. This approach was taken to maintain the robustness of the statistical analyses and avoid biases that could arise from imputation methods. All tests were two-sided, with a significance level set at p < 0.05. The goodness-of-fit of the logistic regression models was assessed using the Hosmer-Lemeshow test, and the variance inflation factor was calculated to check for multicollinearity.

Ethical approval

The study was conducted in accordance with the ethical standards of the institutional and/or national research committee and with the 1964 Helsinki Declaration and its later amendments or comparable ethical standards. Institutional Review Board approval was waived because the study was carried out on a national database that contained de-identified data and did not require informed consent or direct patient participation.

## Results

From 1968 to 2022, the total number of firearm-related deaths in the United States displayed a generally increasing trend, rising from 23,875 in 1968 to 48,205 in 2022. Over the past 55 years, firearm-related suicides consistently accounted for a significant proportion of firearm-related deaths, rising from 10,911 in 1968 to 27,034 in 2022. In contrast, firearm-related homicides, after a decline in the early 2000s, saw a resurgence, climbing from 11,208 in 2013 to 19,645 in 2022 (see Table 1).

**Table 1:** Annual firearm-related deaths in the United States (1968-2022)

	1968	1973	1978	1983	1988	1993	1998	2003	2008	2013	2018	2022
All firearm-related deaths	23,875	31,024	31,635	31,009	33,989	39,595	30,708	30,136	31,593	33,636	39,740	48,204
Firearm-related suicides	10,911	13,317	15,387	16,600	18,169	18,940	17,424	16,907	18,223	21,175	24,432	27,032
Firearm-related homicides	9,425	13,752	13,386	12,040	13,645	18,253	11,798	11,920	12,179	11,208	13,958	19,651

**Figure 1:**
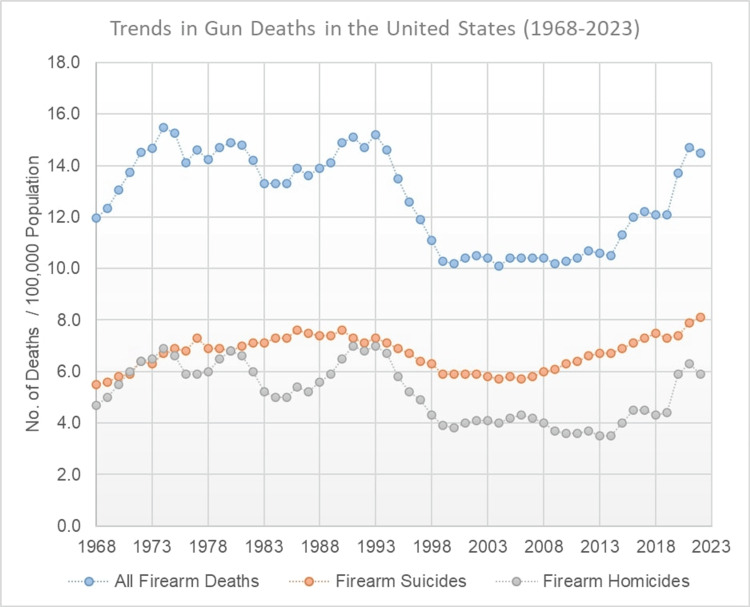
Trends in firearm-related deaths (1968-2022)

**Figure 2:**
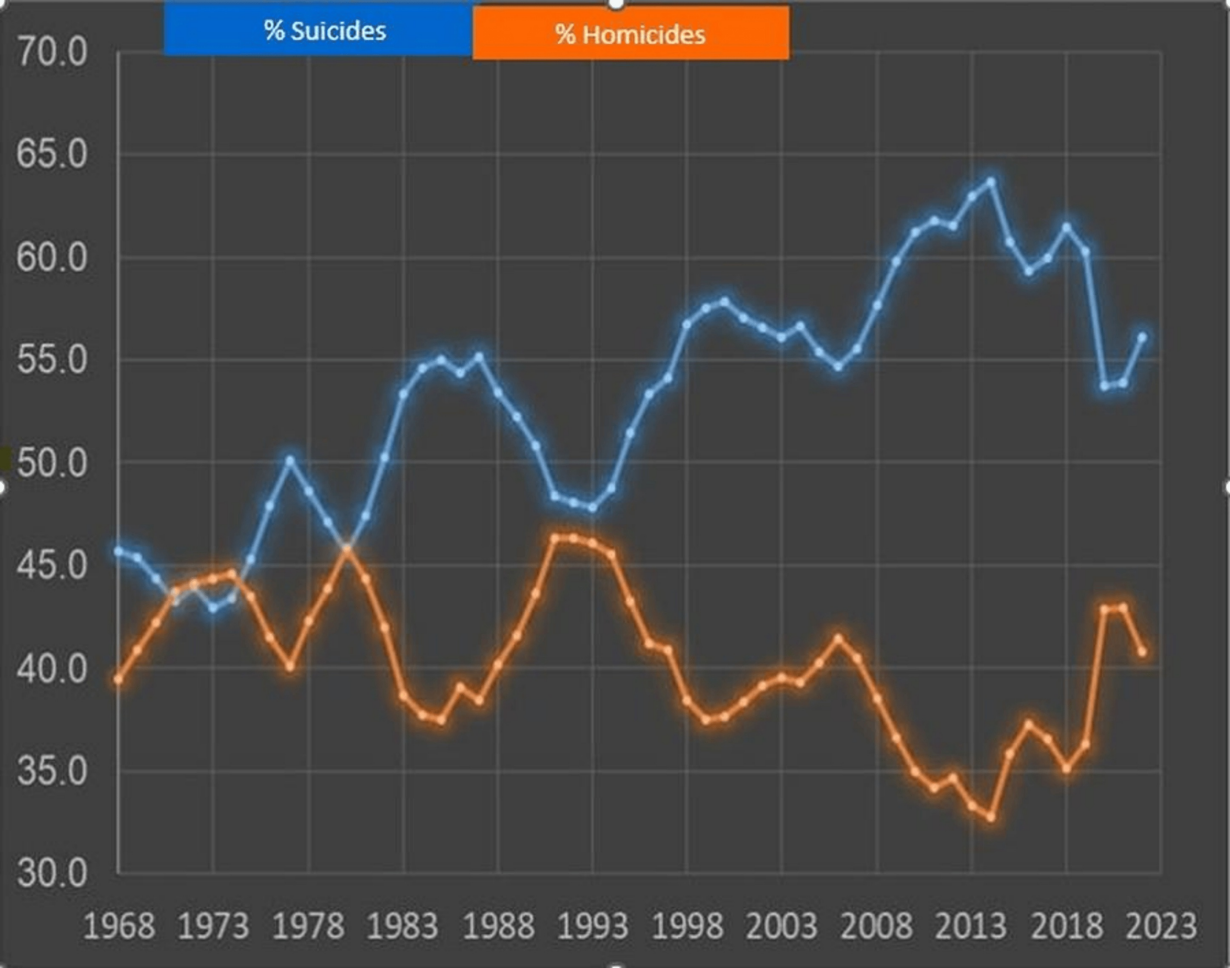
Relative contribution of suicides and homicides to firearm-related deaths in the United States (1968-2022)

Accounting for population growth, we calculated the prevalence of firearm-related deaths per 100,000 population over the past 55 years (see Figure 1). Overall, firearm-related deaths increased from 12.0/100,000 in 1968 to a peak of 15.2/100,000 in 2013, then steadily declined to 10.4/100,000 in 2008 before climbing again to 14.5/100,000 in 2022. In the same period, firearm-related suicides steadily increased from 5.5/100,000 in 1968 to 8.1/100,000 in 2022. Firearm-related homicides increased from 4.4/100,000 in 1968, peaked at 7.0/100,000 in 1993, and then steadily declined to a low of 3.5/100,000 in 2013 before climbing again to 5.9/100,000 in 2022. Over the past 55 years, the proportion of firearm-related deaths due to suicide steadily increased from 45.7% in 1968 to 56.1% in 2022, with a peak of 63% in 2013. On the other hand, the proportion of firearm-related deaths due to homicide remained relatively stable, from 39.5% in 1968 to 40.8%, with the highest proportion of 46.1% in 1993. However, since 2013, there has been a slight upward trend in the proportion of firearm-related deaths due to homicide (see Figure 2).

In a sub-analysis of data (see Table 2) available from 2015 to 2020, which included a total of 186,357 firearm-related deaths, we conducted a multivariable logistic regression analysis with firearm-related suicides and homicides as the independent variables. The analyses were limited to these specific time periods because data on the variables of interest were only available for those years. Covariates in the model included age, gender, education level, race/ethnicity, presence of mental health conditions, substance use disorders, presence of chronic medical conditions, and season of death occurrence. The model also included interaction terms, for example, race/ethnicity x education, to investigate the intersectionality of race/ethnicity and socioeconomic status (using education level as a proxy measure). The goodness-of-fit of the logistic regression models was assessed using the Hosmer-Lemeshow test with p < 0.05, which indicated a good fit. Sensitivity analyses were also conducted using alternative model specifications by removing interaction terms. The results remained consistent across these different scenarios, confirming the stability of our findings.

**Table 2:** Multivariate analysis of independent predictors of firearm-related deaths in the United States by intent (2015-2020)

Variables (N = 186,357; 100%)	Suicides (N = 107,850; 57.9%)	Homicides (N = 74,606; 40.0%)	Unintentional injuries (N = 2,263; 1.2%)
Age			
20-44 years	Reference	Reference	Reference
10-19 years	3.04 (2.92-3.16)	2.87 (2.77-2.97)	6.18 (5.34-7.16)
45-64 years	0.11 (0.110-0.14)	0.04 (0.037-0.038)	0.07 (0.06-0.08)
>64 years	0.02 (0.021-0.023)	0.003 (0.002-0.003)	0.01 (0.008-0.012)
Female	0.19 (0.18-0.19)	0.26 (0.26-0.27)	0.16 (0.14-0.19)
Season			
Winter	Reference	Reference	Reference
Spring	1.02 (1.00-1.07)	1.04 (1.01-1.07)	1.02 (0.87-1.21)
Summer	1.12 (1.09-1.14)	1.27 (1.23-1.31)	1.00 (0.86-1.15)
Fall	0.99 (0.97-1.01)	1.21 (1.18-1.25)	1.00 (0.86-1.15)
Race/ethnicity			
White	Reference	Reference	Reference
Black	0.26 (0.25-0.27)	6.68 (6.50-6.87)	0.92 (0.80-1.06)
Hispanic	0.43 (0.42-0.45)	2.82 (2.73-2.92)	0.69 (0.57-0.84)
Education			
High school	Reference	Reference	Reference
Tertiary	1.42 (1.40-1.45)	0.79 (0.75-0.82)	0.96 (0.83-1.10)
Advanced	1.34 (1.30-1.39)	0.70 (0.63-0.78)	0.58 (0.39-0.86)
Race x education			
White x high school	Reference	Reference	Reference
White x tertiary	1.42 (1.40-1.45)	0.79 (0.75-0.82)	0.96 (0.83-1.10)
White x advanced	1.34 (1.30-1.39)	0.70 (0.63-0.78)	0.58 (0.39-0.86)
Black x high school	0.26 (0.25-0.27)	6.68 (6.50-6.87)	0.92 (0.80-1.06)
Black x tertiary	0.40 (0.38-0.42)	3.85 (3.71-4.00)	0.55 (0.41-0.74)
Black x advanced	0.37 (0.32-0.44)	1.26 (1.06-1.50)	0.28 (0.07-1.13)
Hispanic x high school	0.43 (0.42-0.45)	2.82 (2.73-2.92)	0.69 (0.57-0.84)
Hispanic x tertiary	0.88 (0.83-0.93)	1.87 (1.75-2.00)	0.84 (0.59-1.21)
Hispanic x advanced	0.85 (0.71-1.01)	1.26 (0.94-1.67)	-
Depression	12.9 (12.4-13.5)	0.10 (0.05-0.18)	2.74 (1.57-4.81)
Bipolar disorder	1.61 (1.41-1.84)	0.08 (0.03-0.20)	0.88 (0.22-3.55)
Obesity	0.02 (0.015-0.022)	0.01 (0.005-0.010)	0.04 (0.013-0.12)

The independent risk factors associated with firearm-related suicides include age 10-9 years (OR = 3.04, 95% CI = 2.92-3.16); spring (OR = 1.02, 95% CI = 1.00-1.07) and summer (OR = 1.12, 95% CI = 1.09-1.14) seasons; tertiary (OR = 1.42, 95% CI = 1.40-1.45) and advanced educations (OR = 1.34, 95% CI = 1.30-1.39); depression (OR = 12.9, 95% CI = 12.4-13.5); and bipolar disorder (OR = 1.61, 95% CI = 1.41-1.84). The protective factors associated with firearm-related suicides included obesity (OR = 0.02, 95% CI = 0.015-0.022), female gender (OR = 0.19, 95% CI = 0.18-0.19), Black people (OR = 0.26, 95% CI = 0.25-0.27), and Hispanic people (OR = 0.43, 95% CI = 0.42-0.45). Examining the intersectionality of race/ethnicity and education level, compared to White people with high school degrees, White people with tertiary education (OR = 1.42, 95% CI = 1.40-1.45) and advanced degrees (OR = 1.34, 95% CI = 1.30-1.39) have higher odds of firearm-related suicides, while Black people with high school diploma (OR = 0.26, 95% CI = 0.25-0.27), tertiary degrees (OR = 0.40, 95% CI = 0.38-0.42), and advanced degrees (OR = 0.37, 95% CI = 0.32-0.44) and Hispanic people with high school diploma (OR = 0.43, 95% CI = 0.42-0.45), tertiary degrees (OR = 0.88, 95% CI = 0.83-0.93), and advanced degrees (OR = 0.85, 95% CI = 0.75-1.01) have lower odds of firearm-related suicides. Although they have lower odds of firearm-related suicides, the risk increases with increasing educational attainment, with Black and Hispanic people with college degrees having the highest risk of firearm-related suicides.

Concerning firearm-related homicides, the risk factors identified include age 10-19 years (OR = 2.87, 95% CI = 2.77-2.97); spring (OR = 1.04, 95% CI = 1.01-1.07), summer (OR = 1.27, 95% CI = 1.23-1.31), and fall (OR = 1.21, 95% CI = 1.18-1.25) seasons; Black people (OR = 6.68, 95% CI = 6.50-6.87); and Hispanic people (OR = 2.82, 95% CI = 2.73-2.92). Protective factors include female gender (OR = 0.26, 95% CI = 0.26-0.27); tertiary (OR = 0.79, 95% CI = 0.75-0.82) and advanced (OR = 0.70, 95% CI = 0.63-0.78) degrees; depression (OR = 0.10, 95% CI = 0.05-0.18); bipolar disorder (OR = 0.08, 95% CI = 0.03-0.20); and obesity (OR = 0.01, 95% CI = 0.005-0.010). When examining the intersectionality of race/ethnicity and education level, compared to White people with high school degrees, Black people with high school diploma (OR = 6.68, 95% CI = 6.50-6.87), tertiary education (OR = 3.85, 95% CI = 3.71-4.00), and advanced degrees (OR = 1.26, 95% CI = 1.06-1.50) and Hispanic people with high school diploma (OR = 2.82, 95% CI = 2.73-2.92), tertiary degrees (OR = 1.87, 95% CI = 1.75-2.00), and advanced degrees (OR = 1.26, 95% CI = 0.94-1.67) have higher odds of firearm-related homicides. As the level of education increases for both Black and Hispanic people, the risk of firearm-related homicides decreases.

Risk factors for unintentional injuries include age 10-19 (OR = 6.18, 95% CI = 5.34-7.16), the fall season (OR = 1.21, 95% CI = 1.06-1.38), and depression (OR = 2.74, 95% CI = 1.57-4.81).

## Discussion

In this study, we present trends in firearm-related deaths in the United States over a 55-year period, from 1968 to 2022. Overall, firearm-related deaths have fluctuated but shown a steady increase. A large proportion of these deaths were due to suicide. Firearm-related homicides have steadily decreased since 1993; however, they have begun to rise again in the past decade, with a sharp increase in 2020. The highest risk for firearm-related death occurred among people aged 10-19 years, who have about three times the odds of dying by suicides and homicides and six times the odds of unintentional deaths related to firearms. The highest risk for firearm-related deaths occurred in the summer season, with 27% increased odds for homicides and 12% increased odds for suicides. Highly educated White people have the highest risk for firearm-related suicides, while less-educated Black people have the highest risk for firearm-related homicides. Mental health conditions, especially depression, increase the risk of firearm-related suicides.

Consistent with the existing literature, suicide remains the leading contributor to firearm-related deaths, showing a continuous increase over the past 55 years [3,5,12]. However, unlike mass shootings and homicides, there is less national attention and media sensationalism [3,12]. Indeed, many gun owners are unaware that firearm-related suicides outnumber other firearm-related deaths [13]. Suicide attempts are often method-specific, and many people who are suicidal cannot be assessed or provided with interventions at the time of their highest risk [13]. Therefore, implementing strategies to restrict access to more lethal means is one of the most effective public health interventions to reduce suicide [14,15]. Suicidal ideation is often transient, occurring when protective factors are insufficient and there is acute stress leading to a state of unbearable emotional pain [16]. Access to a lethal firearm, a highly lethal method of suicide, increases the risk of completed suicide attempts [5]. Studies have shown that firearm ownership increases suicide risk [17,18]. Households with guns have a threefold increase in odds of suicides compared to those with no firearms [19]. While having a gun does not increase the prevalence of suicidal ideation, it increases the chances of a fatal outcome if suicidal impulses are acted upon [17-20].

Means restriction targets the population through distal measures and does not depend on an individual’s intention or volition; it has a long history, such as removing carbon monoxide from domestic gas or installing safety fences on bridges [14,21]. Means restriction requires dedicated leadership and enduring political will to be successful [14]. When gun owners are provided with information about firearm-related suicide mortality rates, they have shown a willingness to collaborate on strategies to reduce firearm-related suicides [17]. This underscores the need to engage in public health messaging to inform the public and key stakeholders about firearm restrictions. This means that the conversation around gun control should be about suicide prevention. In reviewing extreme risk protection orders or Red Flag Laws as a restriction methodology, it is evident that lives are saved in jurisdictions with these policies. Data from studies led by the Johns Hopkins School of Public Health suggest that these laws have prevented 21 mass shootings from taking place between 2016 and 2018 [22]. Recent data indicates that 65% of survey participants are aware of these laws, but only 7% are very knowledgeable about them [22].

According to the Health Belief Model, the level of an individual’s perceived vulnerability and perception of the severity of the consequences of a particular condition shapes health behaviors [23]. If individuals understood the increased risk of firearm-related suicides, they might be willing to engage in behaviors to reduce the risks. Similarly, key stakeholders and policymakers need to be engaged to enact policies that restrict access to firearms. Unless firearm restriction is perceived as an effective public health intervention similar to its effectiveness in safeguarding public health and safety as quarantine and vaccinations, enacting and implementing effective nationwide policies will be impossible. This is important given the increasing number of new owners of guns in the United States since 2020 [20]. There is evidence that stronger firearm laws are associated with a reduction in firearm-related suicides without a concomitant increase in suicide by other methods [20,24,25]. However, there are wide variations among states, which makes a nationwide firearm law an essential next step in the prevention of firearm-related suicides [20].

In addition to policy changes, engaging in community-based prevention efforts can have a significant impact on reducing firearm-related suicides. Suicides often increase following the recent purchase of a firearm. One important approach to reducing firearm-related suicides is training firearm retailers to identify customers who may be experiencing a crisis and connect them with appropriate assistance. In addition, disseminating public health messages about the importance of safe firearm storage and how to intervene with someone in a suicide crisis is crucial [17,26,27]. Clinicians also play a crucial role by screening for gun ownership, providing education about safety storage, and identifying at-risk groups (such as those who are acutely suicidal; those with sociodemographic and clinical risk factors for suicide, e.g., people with depression or substance use disorders; males; those with prior history of violence; etc.) [12].

In a sub-analysis of data from 2015 to 2020, we found that people aged 10-19 years have the highest risk of firearm-related suicides compared to older age groups. Several studies have reported rising suicide rates among young people in the past decade. Suicide is the third leading cause of death among high school youths, and death from firearms is the leading cause of death among people aged 19 years and younger [3,28-30]. Suicidality, which encompasses suicidal ideation and suicide attempts, is more prevalent in adolescents compared to adults [31]. Compared to other high-income countries, children in the United States are 11 times more likely to die from firearms [3]. Having firearms at home increases the risk of suicide among children and adolescents [12,32]. About 21% of children live in homes where guns are not safely stored [32]. In studies of adolescents with firearm-related suicide, most of the weapons used belonged to a parent or family member [33-35]. Although several studies report increasing firearm-related suicide rates with age [5,12], a drawback of these studies is that they did not account for potential confounding variables. In this study, using a multivariate logistic regression, younger-aged persons had an increased risk of firearm-related suicides. Older people may have increased suicide rates due to the presence of comorbid depression, anxiety, and substance use compared to adolescents. However, a study showed an overall increase in suicidal ideation and suicide attempts in adolescents without depression [36]. Other factors that may increase the risks for suicide include academic, social, and extracurricular stressors, social media, and worry about climate change and other world events [31,37,38]. Prevention of firearm-related suicides among this age group will require more active interventions than the provision of guidance alone [32]. About two-thirds of gun owners in the United States believe that having a gun makes the home safer; however, most do not realize that gun ownership increases the risk of suicides and other firearm injuries in children and adolescents [32]. Changing norms and beliefs about gun possession and perceived safety should be part of a comprehensive public health approach to suicide prevention.

This study explored the intersectionality of race/ethnicity and education level as a proxy for socioeconomic status. We found that highly educated White people have the highest risk for suicides. For other races/ethnicities, there was an increase in suicide risk with increasing levels of education. Previous studies have reported conflicting results regarding the association between suicide risk and educational attainment, with some studies reporting higher suicide risk with lower levels of education [39,40]. In contrast, others have reported increased suicide risk with higher educational attainment [41,42]. Possible explanations for our findings include a mismatch between educational attainment and anticipated socioeconomic benefits from higher educational attainment; more educated people may be more affected by the loss of earnings and wages from economic depression and the pandemic [41,42]. 

Regarding firearm-related homicides, the highest risk was among young people aged 10-19 years, similar to other studies [43,44]. In addition, we found that the risk among Black people with lower education levels was six times higher compared to other White people with lower educational attainment. Racial disparities in homicides have been previously reported [3,5,12,43,44]. However, there has been little effort in terms of public health response to these racial inequalities [44]. Interventions based on classic epidemiological principles have been advocated, which treat guns like virulent pathogens [44]. This approach would require enhanced surveillance similar to that used for infectious disease control, vector control, and the neutralization of harmful agents [44]. Mitchell et al. describe an effective injury surveillance system [45]. Addressing structural racism and other risk factors at the interpersonal and community levels is also essential [43,44]. These factors include improving access to equitable educational opportunities, alleviating poverty, creating opportunities for social connectedness and employment, developing the built environment by providing infrastructures, and providing adequate lighting [46].

As previously reported in extant literature, this study found that depression and bipolar disorder increased the risk of firearm-related suicides [12,47]. Mental health conditions and substance use disorders continue to be significant risk factors for suicides, and improved access to mental health services can reduce individual-level risk factors for firearm-related suicides [47-49]. Screening for firearms among patients with mental health conditions by clinicians is a vital suicide prevention strategy. This should include safety planning around the secure storage of weapons and involve friends and family in these conversations when appropriate [12,49].

Limitations

There are several limitations to consider when interpreting our study. Firstly, this is a cross-sectional study and precludes causal inference. Secondly, our sub-analysis was limited to seven years (the only years data was available for the covariates); therefore, results may have overestimated or underestimated the measures of association reported in this study. Thirdly, our analysis relies on the accuracy and completeness of the data available in WISQARS, and inaccuracies in the data will introduce bias into our study. Fourthly, WISQARS may be subject to underreporting and misclassifying deaths as the data depends on reporting practices of different jurisdictions. Misclassification of deaths could lead to under- or overestimation of our measures of association. Fifthly, WISQARS data focuses mainly on injury-related variables and does not provide comprehensive information on socioeconomic factors that may influence injury patterns and risks. While we have explored intersectionality in our analysis, we are not able to explore the complex interplay between socioeconomic status and firearm-related deaths. Our data used educational attainment as a proxy measure for socioeconomic status, but socioeconomic status is multidimensional. Therefore, our analysis is unable to capture this complexity. Sixthly, there is potential for selection bias as there is the possibility of differential reporting by demographic factors such as age, gender, race/ethnicity, variability in autopsy rates, or incomplete or inaccurate death certification process. Seventh, given the number of predictors and interaction terms analyzed in our multivariate regression model, there is an inherent risk of false-positive findings. We have carefully interpreted our findings to mitigate this risk, emphasizing those with strong statistical significance and theoretical support or clinical relevance. However, we acknowledge this limitation and recommend cautiously interpreting results, particularly those with marginal statistical significance.

## Conclusions

In this study, we aimed to provide insights into the changing patterns and risk and protective factors associated with firearm-related deaths in the United States over the past 55 years. Firearm-related deaths are the leading causes of death in the United States and have continued to rise over the past 55 years. Despite the sensationalism around homicides and mass shootings, firearm-related suicides are significant contributors to firearm-related deaths but do not receive a lot of media attention. Our study shows that individuals aged 10-19 years have the highest risk of firearm-related deaths, both due to suicides and homicides. In addition, highly educated White people and individuals with depression and bipolar disorders have a higher risk of firearm-related suicides.

## References

[1] Gramlich J (2024). Gramlich J: What the data says about gun deaths in the US. Pew Research Center. https://www.pewresearch.org/short-reads/2023/04/26/what-the-data-says-about-gun-deaths-in-the-u-s/.

[2] Grinshteyn E, Hemenway D (2019). Violent death rates in the US compared to those of the other high-income countries, 2015. Prev Med.

[3] Mueller KL, Lovelady NN, Ranney ML (2023). Firearm injuries and death: a United States epidemic with public health solutions. PLOS Glob Public Health.

[4] (2024). Centers for Disease Control and Prevention. The social-ecological model: a framework for prevention. https://www.cdc.gov/violence-prevention/about/index.html.

[5] Sakran JV, Lunardi N (2022). Reducing firearm injury and death in the United States. Adv Surg.

[6] National Research Council (2013). Priorities for Research to Reduce the Threat of Firearm-Related Violence.

[7] Powell RE, Sacks CA (2020). A national research strategy to reduce firearm-related injury and death: recommendations from the Health Policy Research Subcommittee of the Society of General Internal Medicine (SGIM). J Gen Intern Med.

[8] Kellermann AL, Rivara FP (2013). Silencing the science on gun research. JAMA.

[9] Steinwachs DM (2014). Transforming public health systems: using data to drive organizational capacity for quality improvement and efficiency. EGEMS (Wash DC).

[10] Chao K, Sarker MN, Ali I, Firdaus RB, Azman A, Shaed MM (2023). Big data-driven public health policy making: potential for the healthcare industry. Heliyon.

[11] Wilson RF, Liu G, Lyons BH, Petrosky E, Harrison DD, Betz CJ, Blair JM (2022). Surveillance for violent deaths - National Violent Death Reporting System, 42 states, the District of Columbia, and Puerto Rico, 2019. MMWR Surveill Summ.

[12] Pallin R, Spitzer SA, Ranney ML, Betz ME, Wintemute GJ (2019). Preventing firearm-related death and injury. Ann Intern Med.

[13] Rabin RC (2024). The New York Times: Rabin RC: How did we not know?’ Gun owners confront a suicide epidemic. https://www.nytimes.com/2020/11/17/health/suicide-guns-prevention.html.

[14] Yip PS, Caine E, Yousuf S, Chang SS, Wu KC, Chen YY (2012). Means restriction for suicide prevention. Lancet.

[15] Bridges FS (2004). Gun control law (Bill C-17), suicide, and homicide in Canada. Psychol Rep.

[16] Institute of Medicine (US) (2001). Board on Neuroscience and Behavioral Health. Risk Factors for Suicide: Summary of a Workshop.

[17] Walton T, Stuber J (2020). Firearm retailers and suicide: results from a survey assessing willingness to engage in prevention efforts. Suicide Life Threat Behav.

[18] Siegel M, Rothman EF (2016). Firearm ownership and suicide rates among US men and women, 1981-2013. Am J Public Health.

[19] Anglemyer A, Horvath T, Rutherford G (2014). The accessibility of firearms and risk for suicide and homicide victimization among household members: a systematic review and meta-analysis. Ann Intern Med.

[20] Kaufman EJ, Morrison CN, Branas CC, Wiebe DJ (2018). State firearm laws and interstate firearm deaths from homicide and suicide in the United States: a cross-sectional analysis of data by county. JAMA Intern Med.

[21] Pelletier AR (2007). Preventing suicide by jumping: the effect of a bridge safety fence. Inj Prev.

[22] Magee F (2024). Magee F: Research indicates that red flag laws work - but only if people know about them. https://www.thetrace.org/2023/08/extreme-risk-protection-order-laws-study/.

[23] Champion VL, Skinner CS (2015). The health belief model. Health Behavior, Theory and Research.

[24] Crifasi CK, Meyers JS, Vernick JS, Webster DW (2015). Effects of changes in permit-to-purchase handgun laws in Connecticut and Missouri on suicide rates. Prev Med.

[25] Anestis MD, Khazem LR, Law KC, Houtsma C, LeTard R, Moberg F, Martin R (2015). The association between state laws regulating handgun ownership and statewide suicide rates. Am J Public Health.

[26] Vriniotis M, Barber C, Frank E, Demicco R (2015). A suicide prevention campaign for firearm dealers in New Hampshire. Suicide Life Threat Behav.

[27] Wintemute GJ, Parham CA, Beaumont JJ, Wright M, Drake C (1999). Mortality among recent purchasers of handguns. N Engl J Med.

[28] Gaylor EM, Krause KH, Welder LE (2023). Suicidal thoughts and behaviors among high school students - youth risk behavior survey, United States, 2021. MMWR Suppl.

[29] Van Meter AR, Knowles EA, Mintz EH (2023). Systematic review and meta-analysis: international prevalence of suicidal ideation and attempt in youth. J Am Acad Child Adolesc Psychiatry.

[30] Farah R, Rege SV, Cole RJ, Holstege CP (2023). Suspected suicide attempts by self-poisoning among persons aged 10-19 years during the COVID-19 pandemic - United States, 2020-2022. MMWR Morb Mortal Wkly Rep.

[31] Kim Y, Krause TM, Lane SD (2023). Trends and seasonality of emergency department visits and hospitalizations for suicidality among children and adolescents in the US from 2016 to 2021. JAMA Netw Open.

[32] Azrael D, Cohen J, Salhi C, Miller M (2018). Firearm storage in gun-owning households with children: results of a 2015 national survey. J Urban Health.

[33] Johnson RM, Barber C, Azrael D, Clark DE, Hemenway D (2010). Who are the owners of firearms used in adolescent suicides?. Suicide Life Threat Behav.

[34] Wright MA, Wintemute GJ, Claire BE (2008). Gun suicide by young people in California: descriptive epidemiology and gun ownership. J Adolesc Health.

[35] Grossman DC, Reay DT, Baker SA (1999). Self-inflicted and unintentional firearm injuries among children and adolescents: the source of the firearm. Arch Pediatr Adolesc Med.

[36] Han B, Compton WM, Blanco C, Colpe L, Huang L, McKeon R (2018). National trends in the prevalence of suicidal ideation and behavior among young adults and receipt of mental health care among suicidal young adults. J Am Acad Child Adolesc Psychiatry.

[37] Ciccotti HR, Spiller HA, Casavant MJ, Kistamgari S, Funk AR, Smith GA (2023). Pediatric suspected suicides and nonfatal suicide attempts reported to United States Poison Control Centers before and during the COVID-19 pandemic. J Med Toxicol.

[38] Sheridan DC, Grusing S, Marshall R, Lin A, Hughes AR, Hendrickson RG, Horowitz BZ (2022). Changes in suicidal ingestion among preadolescent children from 2000 to 2020. JAMA Pediatr.

[39] Denney JT, Rogers RG, Krueger PM, Wadsworth T (2009). Adult suicide mortality in the United States: marital status, family size, socioeconomic status, and differences by sex. Soc Sci Q.

[40] Øien-Ødegaard C, Hauge LJ, Reneflot A (2021). Marital status, educational attainment, and suicide risk: a Norwegian register-based population study. Popul Health Metr.

[41] Shah A, Chatterjee S (2008). Is there a relationship between elderly suicide rates and educational attainment? A cross-national study. Aging Ment Health.

[42] Pompili M, Vichi M, Qin P, Innamorati M, De Leo D, Girardi P (2013). Does the level of education influence completed suicide? A nationwide register study. J Affect Disord.

[43] Pear VA, Wintemute GJ, Jewell NP, Cerdá M, Ahern J (2023). Community-level risk factors for firearm assault and homicide: the role of local firearm dealers and alcohol outlets. Epidemiology.

[44] Levine RS, Goldzweig I, Kilbourne B, Juarez P (2012). Firearms, youth homicide, and public health. J Health Care Poor Underserved.

[45] Mitchell RJ, Williamson AM, O’Connor R (2009). The development of an evaluation framework for injury surveillance systems. BMC Public Health.

[46] World Bank. Violence in the city: understanding and supporting community responses to urban violence. https://documents.worldbank.org/en/publication/documents-reports/documentdetail/524341468331181450/violence-in-the-city-understanding-and-supporting-community-responses-to-urban-violence.

[47] Swanson JW, McGinty EE, Fazel S, Mays VM (2015). Mental illness and reduction of gun violence and suicide: bringing epidemiologic research to policy. Ann Epidemiol.

[48] Wintemute GJ (2011). Association between firearm ownership, firearm-related risk and risk reduction behaviours and alcohol-related risk behaviours. Inj Prev.

[49] Li Z, Page A, Martin G, Taylor R (2011). Attributable risk of psychiatric and socio-economic factors for suicide from individual-level, population-based studies: a systematic review. Soc Sci Med.

